# Weighted *A*-Statistical Convergence for Sequences of Positive Linear Operators

**DOI:** 10.1155/2014/437863

**Published:** 2014-07-01

**Authors:** S. A. Mohiuddine, Abdullah Alotaibi, Bipan Hazarika

**Affiliations:** ^1^Department of Mathematics, Faculty of Science, King Abdulaziz University, P.O. Box 80203, Jeddah 21589, Saudi Arabia; ^2^Department of Mathematics, Rajiv Gandhi University, Rono Hills, Doimukh, Arunachal Pradesh 791 112, India

## Abstract

We introduce the notion of weighted *A*-statistical convergence of a sequence, where *A* represents the nonnegative regular matrix. We also prove the Korovkin approximation theorem by using the notion of weighted *A*-statistical convergence. Further, we give a rate of weighted *A*-statistical convergence and apply the classical Bernstein polynomial to construct an illustrative example in support of our result.

## 1. Background, Notations, and Preliminaries

We begin this paper by recalling the definition of natural (or asymptotic) density as follows. Suppose that *E*⊆*N* : = {1,2,…} and *E*_*n*_ = {*k* ≤ *n* : *k* ∈ *E*}. Then

(1) $$\begin{array}{c} \delta \left(E\right)=\underset{n}{\lim}\frac{1}{n}\left|E_{n}\right| \end{array}$$

is called the* natural density* of *E* provided that the limit exists, where |·| represents the number of elements in the enclosed set.

The term “statistical convergence” was first presented by Fast [1] which is a generalization of the concept of ordinary convergence. Actually, a root of the notion of statistical convergence can be detected by Zygmund [2] (also, see [3]), where he used the term “almost convergence” which turned out to be equivalent to the concept of statistical convergence. The notion of Fast was further investigated by Schoenberg [4], Šalát [5], Fridy [6], and Conner [7].

The following notion is due to Fast [1]. A sequence *x* = (*x*_*k*_) is said to be* statistically convergent* to *L* if *δ*(*K*_*ϵ*_) = 0 for every *ϵ* > 0, where

(2) $$\begin{array}{c} K_{\epsilon }:=\left\{k\in N:\left|x_{k}-L\right|\geq \epsilon \right\}. \end{array}$$

Equivalently,

(3) $$\begin{array}{c} \underset{n}{\lim}\;n^{-1}\left|\left\{k\leq n:\left|x_{k}-L\right|\geq \epsilon \right\}\right|=0. \end{array}$$

In symbol, we will write *S*-lim *x* = *L*. We remark that every convergent sequence is statistically convergent but not conversely.

Let *X* and *Y* be two sequence spaces and let *A* = (*a*_*n*,*k*_) be an infinite matrix. If for each *x* = (*x*_*k*_) in *X* the series

(4) $$\begin{array}{c} A_{n}x=\underset{k}{\sum }a_{n,k}x_{k}=\overset{\infty }{\underset{k=1}{\sum }}a_{n,k}x_{k} \end{array}$$

converges for each *n* ∈ *N* and the sequence *Ax* = (*A*_*n*_ *x*) belongs to *Y*, then we say that matrix *A* maps *X* into *Y*. By the symbol (*X*, *Y*) we denote the set of all matrices which map *X* into *Y*.

A matrix *A* (or a matrix map *A*) is called regular if *A* ∈ (*c*, *c*), where the symbol *c* denotes the spaces of all convergent sequences and

(5) $$\begin{array}{c} \underset{n}{\lim}\;A_{n}x=\underset{k}{\lim}\;x_{k} \end{array}$$

for all *x* ∈ *c*. The well-known Silverman-Toeplitz theorem (see [8]) asserts that *A* = (*a*_*n*,*k*_) is regular if and only if

- lim⁡_*n*_ *a*_*n*,*k*_ = 0 for each *k*;
- lim⁡_*n*_∑_*k*_ *a*_*n*,*k*_ = 1;
- sup⁡_*n*_∑_*k*_ | *a*_*n*,*k*_ | <*∞*.

Kolk [9] extended the definition of statistical convergence with the help of nonnegative regular matrix *A* = (*a*_*n*,*k*_) calling it *A*-statistical convergence. The definition of *A*-statistical convergence is given by Kolk as follows. For any nonnegative regular matrix *A*, we say that a sequence is *A*-statistically convergent to *L* provided that for every *ϵ* > 0 we have

(6) $$\begin{array}{c} \underset{n}{\lim}\underset{k:|x_{n}-L|\geq \epsilon }{\sum }a_{n,k}=L. \end{array}$$

In 2009, the concept of weighted statistical convergence was defined and studied by Karakaya and Chishti [10] and further modified by Mursaleen et al. [11] in 2012. In 2013, Belen and Mohiuddine [12] presented a generalization of this notion through de la Vallée-Poussin mean. Quite recently, Esi [13] defined and studied the notion statistical summability through de la Vallée-Poussin mean in probabilistic normed spaces.

Let *p* = (*p*_*k*_) be a sequence of nonnegative numbers such that *p*_0_ > 0 and

(7) $$\begin{array}{c} P_{n}=\overset{n}{\underset{k=0}{\sum }}p_{k}⟶\infty \;\text{as}\;n⟶\infty . \end{array}$$

Let

(8) $$\begin{array}{c} t_{n}=\frac{1}{P_{n}}\overset{n}{\underset{k=0}{\sum }}p_{k}x_{k},\;n=0,1,2\ldots . \end{array}$$

We say that the sequence *x* = (*x*_*k*_) is $\left(\bar{N},p_{n}\right)$-*summable* to *L* if lim⁡_*n*→*∞*_ *t*_*n*_ = *L*.

The lower and upper weighted densities of *E*⊆*N* are defined by

(9) $$\begin{array}{c} {\underline{\delta }}_{\bar{N}}\left(E\right)=\underset{n}{\mathrm{liminf}}\frac{1}{P_{n}}\left|\left\{k\leq P_{n}:k\in E\right\}\right|, \end{array}$$

(10) $$\begin{array}{c} {\bar{\delta }}_{\bar{N}}\left(E\right)=\underset{n}{\mathrm{limsup}}\frac{1}{P_{n}}\left|\left\{k\leq P_{n}:k\in E\right\}\right|, \end{array}$$

respectively. We say that *E* has* weighted density*, denoted by $\delta_{\bar{N}}(E)$, if the limits of both of the above densities exist and are equal; that is, one writes

(11) $$\begin{array}{c} \delta_{\bar{N}}\left(E\right)=\underset{n}{\lim}\frac{1}{P_{n}}\left|\left\{k\leq P_{n}:k\in E\right\}\right|. \end{array}$$

The sequence *x* = (*x*_*k*_) is said to be* weighted statistically convergent* (or $S_{\bar{N}}\text{-}convergent$) to *L* if, for every *ϵ* > 0, the set {*k* ∈ *N* : *p*_*k*_ | *x*_*k*_ − *L* | ≥*ϵ*} has weighted density zero; that is,

(12) $$\begin{array}{c} \underset{n}{\lim}\frac{1}{P_{n}}\left|\left\{k\leq P_{n}:p_{k}\left|x_{k}-L\right|\geq \epsilon \right\}\right|=0. \end{array}$$

In this case we write $L=S_{\bar{N}}\text{-lim}\;x$.

**Remark 1 .**

If *p*_*k*_ = 1 for all *k*, then $\left(\bar{N},p_{n}\right)$-summable is reduced to (*C*, 1)-summable (or Cesàro summable) and weighted statistical convergence is reduced to statistical convergence.

On the other hand, let us recall that *C*[*a*, *b*] is the space of all functions *f* continuous on [*a*, *b*]. We know that *C*[*a*, *b*] is a Banach space with norm

(13) $$\begin{array}{c} {||f||}_{\infty }:=\underset{x\in [a,b]}{\sup}\left|f\left(x\right)\right|,\;f\in C\left[a,b\right]. \end{array}$$

Suppose that *L* is a linear operator from *C*[*a*, *b*] into *C*[*a*, *b*]. It is clear that if *f* ≥ 0 implies *Lf* ≥ 0, then the linear operator *L* is positive on *C*[*a*, *b*]. We denote the value of *Lf* at a point *x* ∈ [*a*, *b*] by *L*(*f*; *x*). The classical Korovkin approximation theorem states the following [14].

**Theorem 2 .**

Let (*T*_*n*_) be a sequence of positive linear operators from *C*[*a*, *b*] into *C*[*a*, *b*]. Then,

(14) $$\begin{array}{c} \underset{n}{\lim}\;{||T_{n}(f,x)-f(x)||}_{\infty }=0, \end{array}$$

for all *f* ∈ *C*[*a*, *b*] if and only if

(15) $$\begin{array}{c} \underset{n}{\lim}\;{||T_{n}(f_{i},x)-f_{i}(x)||}_{\infty }=0, \end{array}$$

where *f*_*i*_(*x*) = *x* ^*i*^ and *i* = 0,1, 2.

Many mathematicians extended the Korovkin-type approximation theorems by using various test functions in several setups, including Banach spaces, abstract Banach lattices, function spaces, and Banach algebras. Firstly, Gadjiev and Orhan [15] established classical Korovkin theorem through statistical convergence and display an interesting example in support of our result. Recently, Korovkin-type theorems have been obtained by Mohiuddine [16] for almost convergence. Korovkin-type theorems were also obtained in [17] for *λ*-statistical convergence. The authors of [18] established these types of approximation theorem in weighted *L*_*p*_ spaces, where 1 ≤ *p* < *∞*, through *A*-summability which is stronger than ordinary convergence. For these types of approximation theorems and related concepts, one can be referred to [19–27] and references therein.

## 2. Korovkin-Type Theorems by Weighted *A*-Statistical Convergence

Kolk [9] introduced the notion of *A*-statistical convergence by considering nonnegative regular matrix *A* instead of Cesáro matrix in the definition of statistical convergence due to Fast. Inspired from the work of Kolk, we introduce the notion of weighted *A*-statistical convergence of a sequence and then we establish some Korovkin-type theorems by using this notion.

**Definition 3 .**

Let *A* = (*a*_*n*,*k*_) be a nonnegative regular matrix. A sequence *x* = (*x*_*k*_) of real or complex numbers is said to be* weighted A-statistically convergent*, denoted by $S_{A}^{\bar{N}}$-convergent, to *L* if for every *ϵ* > 0

(16) $$\begin{array}{c} \underset{n}{\lim}\underset{k\in E(p,\epsilon )}{\sum }a_{n,k}=0, \end{array}$$

where

(17) $$\begin{array}{c} E\left(p,\epsilon \right)=\left\{k\in N:p_{k}\left|x_{k}-L\right|\geq \epsilon \right\}. \end{array}$$

In symbol, we will write $S_{A}^{\bar{N}}\text{-lim}\;x=L$.

**Remark 4 .**

One has the following.

- If we take *A* = *I*, where *I* denotes the identity matrix, then weighted *A*-statistical convergence of a sequence is reduced to ordinary convergence.
- If we take *A* = (*C*, 1), where (*C*, 1) denotes the Cesáro matrix of order one, then weighted *A*-statistical convergence of a sequence reduces to weighted statistical convergence.
- If we take *A* = (*C*, 1) and *p*_*k*_ = 1 for all *k*, then weighted *A*-statistical convergence of a sequence reduces to statistical convergence.

Note that convergent sequence implies weighted *A*-statistically convergent to the same value but the converse is not true in general. For example, take *A* = (*C*, 1) and *p*_*k*_ = 1 for all *k* and define a sequence *x* = (*x*_*k*_) by

(18) $$\begin{array}{c} x_{k}=\{\begin{array}{cc} 1, & \text{if}\;k=n^{2},\\ 0, & \text{otherwise}, \end{array} \end{array}$$

where *n* ∈ *N*. Then this sequence is statistically convergent to 0 but not convergent; in this case, weighted *A*-statistical convergence of a sequence coincides with statistical convergence.

**Theorem 5 .**

Let *A* = (*a*_*n*,*k*_) be a nonnegative regular matrix. Consider a sequence of positive linear operators (*M*_*k*_) from *C*[*a*, *b*] into itself. Then, for all *f* ∈ *C*[*a*, *b*] bounded on whole real line,

(19) $$\begin{array}{c} S_{A}^{\bar{N}}\text{-}\underset{k}{\lim}{||M_{k}\left(f,x\right)-f(x)||}_{\infty }=0 \end{array}$$

if and only if

(20) $$\begin{array}{c} S_{A}^{\bar{N}}\text{-}\underset{k}{\lim}{||M_{k}\left(1,x\right)-1||}_{\infty }=0,\\ S_{A}^{\bar{N}}\text{-}\underset{k}{\lim}{||M_{k}\left(v,x\right)-x||}_{\infty }=0,\\ S_{A}^{\bar{N}}\text{-}\underset{k}{\lim}{||M_{k}\left(v^{2},x\right)-x^{2}||}_{\infty }=0. \end{array}$$

**Proof**

Equation (20) directly follows from (19) because each of 1, *x*, *x* ^2^ belongs to *C*[*a*, *b*]. Consider a function *f* ∈ *C*[*a*, *b*]. Then there is a constant *C* > 0 such that |*f*(*x*)| ≤ *C* for all *x* ∈ (−*∞*, *∞*). Therefore,

(21) $$\begin{array}{c} |f\left(v\right)-f\left(x\right)|\leq 2C,\;-\infty <v,\;x<\infty . \end{array}$$

Let *ϵ* > 0 be given. By hypothesis there is a *δ* : = *δ*(*ɛ*) > 0 such that

(22) $$\begin{array}{c} |f\left(v\right)-f\left(x\right)|<\epsilon \;\forall \left|v-x\right|<\delta . \end{array}$$

Solving (21) and (22) and then substituting Ω(*v*) = (*v* − *x*)^2^, one obtains

(23) $$\begin{array}{c} \left|f\left(v\right)-f\left(x\right)\right|<\epsilon +\frac{2C}{\delta^{2}}\Omega ,\;\forall \left|v-x\right|<\delta . \end{array}$$

Equation (23) can also be written as

(24) $$\begin{array}{c} -\epsilon -\frac{2C}{\delta^{2}}\Omega <f\left(v\right)-f\left(x\right)<\epsilon +\frac{2M}{\delta^{2}}\Omega . \end{array}$$

Operating *M*_*k*_(1, *x*) to (24) since *M*_*k*_(*f*, *x*) is linear and monotone, one obtains

(25) Mk(1,x)(−ϵ−2Cδ2Ω)<Mk(1,x)(f(v)−f(x))<Mk(1,x)(ϵ+2Cδ2Ω).

Note that *x* is fixed, so *f*(*x*) is constant number. Thus, we obtain from (25) that

(26) −ϵMk(1,x)−2Cδ2Mk(Ω,x)<Mk(f,x)−f(x)Mk(1,x)<ϵMk(1,x)+2Cδ2Mk(Ω,x).

The term “*M*_*k*_(*f*, *x*) − *f*(*x*)*M*_*k*_(1, *x*)” in (26) can also be written as

(27) Mk(f,x)−f(x)Mk(1,x)=Mk(f,x)−f(x)−f(x)[Mk(1,x)−1].

Now substituting the value of *M*_*k*_(*f*, *x*) − *f*(*x*)*M*_*k*_(1, *x*) in (26), we get that

(28) Mk(f,x)−f(x)<ϵMk(1,x)+2Cδ2Mk(Ω,x)+f(x)(Mk(1,x)−1).

We can rewrite the term “*M*_*k*_(Ω, *x*)” in (28) as follows:

(29) Mk(Ω,x)=Mk((v−x)2,x)=Mk(v2,x)+2xMk(v,x)+x2Mk(1,x)=[Mk(v2,x)−x2]−2x[Mk(v,x)−x]+x2[Mk(1,x)−1].

Equation (28) with the above value of *M*_*k*_(Ω, *x*) becomes

(30) Mk(f,x)−f(x) <ϵMk(1,x)  +2Cδ2{[Mk(v2,x)−x2]+2x[Mk(v,x)−x]     + x2[Mk(1,x)−1]}+f(x)(Mk(1,x)−1) =ϵ[Mk(1,x)−1]+ϵ  +2Cδ2{[Mk(v2,x)−x2]+2x[Mk(v,x)−x]     + x2[Mk(1,x)−1]}+f(x)[Mk(1,x)−1].

Therefore,

(31) |Mk(f,x)−f(x)|≤(ϵ+2Cb2δ2+C)|Mk(1,x)−1|+2Cδ2|Mk(v2,x)−x2|+4Cbδ2|Mk(v,x)−x|,

where *b* = max⁡|*x*|. Taking supremum over *x* ∈ [*a*, *b*], one obtains

(32) ||Mk(f,x)−f(x)||∞≤(ϵ+2Cb2δ2+C)||Mk(1,x)−1||∞+2Cδ2||Mk(v2,x)−x2||∞+4Cbδ2||Mk(v,x)−x||∞,

or

(33) ||Mk(f,x)−f(x)||∞ ≤T{||Mk(1,x)−1||∞+||Mk(v2,x)−x2||∞     + ||Mk(v,x)−x||∞},

where

(34) $$\begin{array}{c} T:=\max\left\{\epsilon +\frac{2Cb^{2}}{\delta^{2}}+C,\frac{2C}{\delta^{2}},\frac{4Cb}{\delta^{2}}\right\}. \end{array}$$

Hence,

(35) pk||Mk(f,x)−f(x)||∞ ≤T{pk||Mk(1,x)−1||∞+pk||Mk(v2,x)−x2||∞    + pk||Mk(v,x)−x||∞}.

For a given *α* > 0, choose *ϵ* > 0 such that *ϵ* < *α*, and we will define the following sets:

(36) E={k∈N:pk||Mk(f,x)−f(x)||∞≥α},E1={k∈N:pk||Mk(1,x)−1||∞≥α−ϵ3T},E2={k∈N:pk||Mk(v,x)−x||∞≥α−ϵ3T},E3={k∈N:pk||Mk(v2,x)−x2||∞≥α−ϵ3T}.

It is easy to see that

(37) $$\begin{array}{c} E\subset E_{1}\cup E_{2}\cup E_{3}. \end{array}$$

Thus, for each *n* ∈ *N*, we obtain from (35) that

(38) $$\begin{array}{c} \underset{k\in E}{\sum }a_{n,k}\leq \underset{k\in E_{1}}{\sum }a_{n,k}+\underset{k\in E_{2}}{\sum }a_{n,k}+\underset{k\in E_{3}}{\sum }a_{n,k}. \end{array}$$

Taking limit *n* → *∞* in (38) and also (20) gives that

(39) $$\begin{array}{c} \underset{n}{\lim}\underset{k\in E}{\sum }a_{n,k}=0. \end{array}$$

This yields that

(40) $$\begin{array}{c} S_{A}^{\bar{N}}\text{-}\underset{k}{\lim}{||M_{k}(f,x)-f(x)||}_{\infty }=0, \end{array}$$

for all *f* ∈ *C*[*a*, *b*].

We also obtain the following Korovkin-type theorem for weighted statistical convergence by writing Cesáro matrix (*C*, 1) instead of nonnegative regular matrix *A* in Theorem 5.

**Theorem 6 .**

Consider a sequence of positive linear operators (*M*_*k*_) from *C*[*a*, *b*] into itself. Then, for all *f* ∈ *C*[*a*, *b*]

(41) $$\begin{array}{c} S_{\bar{N}}\text{-}\underset{k}{\lim}\;{||M_{k}\left(f,x\right)-f(x)||}_{\infty }=0 \end{array}$$

if and only if

(42) $$\begin{array}{c} S_{\bar{N}}\text{-}\underset{k}{\lim}\;{||M_{k}\left(1,x\right)-1||}_{\infty }=0, \end{array}$$

(43) $$\begin{array}{c} S_{\bar{N}}\text{-}\underset{k}{\lim}\;{||M_{k}\left(v,x\right)-x||}_{\infty }=0, \end{array}$$

(44) $$\begin{array}{c} S_{\bar{N}}\text{-}\underset{k}{\lim}\;{||M_{k}\left(v^{2},x\right)-x^{2}||}_{\infty }=0. \end{array}$$

**Proof**

Following the proof of Theorem 5, one obtains

(45) $$\begin{array}{c} E\subset E_{1}\cup E_{2}\cup E_{3} \end{array}$$

and so

(46) $$\begin{array}{c} \delta_{\bar{N}}\left(E\right)\subset \delta_{\bar{N}}\left(E_{1}\right)+\delta_{\bar{N}}\left(E_{2}\right)+\delta_{\bar{N}}\left(E_{3}\right). \end{array}$$

Equations (42)–(44) give that

(47) $$\begin{array}{c} S_{\bar{N}}\text{-}\underset{k}{\lim}\;{||M_{k}\left(f,x\right)-f(x)||}_{\infty }=0. \end{array}$$

**Remark 7 .**

If we replace nonnegative regular matrix *A* by Cesáro matrix and choose *p*_*k*_ = 1 for all *k*, in Theorem 5, then we obtain Theorem 1 due to Gadjiev and Orhan [15].

**Remark 8 .**

By Theorem 2 of [10], we have that if a sequence *x* = (*x*_*k*_) is weighted statistically convergent to *L*, then it is strongly $\left(\bar{N},p_{n}\right)$-summable to *L* provided that *p*_*k*_ | *x*_*k*_ − *L*| is bounded; that is, there exists a constant *C* such that *p*_*k*_ | *x*_*k*_ − *L* | ≤*C* for all *k* ∈ *N*. We write

(48) $$\begin{array}{c} |\bar{N},p_{n}|=\left\{x=\left(x_{k}\right):\underset{n}{\lim}\frac{1}{P_{n}}\overset{n}{\underset{k=0}{\sum }}p_{k}\left|x_{k}-L\right|=0\;\text{for}\;\text{some}\;L\right\} \end{array}$$

for the set of all sequences *x* = (*x*_*k*_) which are strongly $\left(\bar{N},p_{n}\right)$-summable to *L*.

**Theorem 9 .**

Let *M*_*k*_ : *C*[*a*, *b*] → *C*[*a*, *b*] be a sequence of positive linear operators which satisfies (43)-(44) of Theorem 6 and the following condition holds:

(49) $$\begin{array}{c} \underset{k}{\lim}\;{||M_{k}(1,x)-1||}_{\infty }=0. \end{array}$$

Then,

(50) $$\begin{array}{c} \underset{n}{\lim}\frac{1}{P_{n}}\overset{n}{\underset{k=0}{\sum }}p_{k}\;{||M_{k}(f,x)-f(x)||}_{\infty }=0, \end{array}$$

for any *f* ∈ *C*[*a*, *b*].

**Proof**

It follows from (49) that ||*M*_*k*_(1,*x*)||_*∞*_ ≤ *C*′, for some constant *C*′ > 0 and for all *k* ∈ *N*. Hence, for *f* ∈ *C*[*a*, *b*], one obtains

(51) pk||Mk(f,x)−f(x)||∞≤pk(||f||∞||Mk(1,x)||∞+||f||∞)≤pkC(C′+1).

Right hand side of (51) is constant, so *p*_*k*_||*M*_*k*_(*f*,*x*)−*f*(*x*)||_*∞*_ is bounded. Since (49) implies (42), by Theorem 6 we get that

(52) $$\begin{array}{c} S_{\bar{N}}\text{-lim}{||M_{k}(f,x)-f(x)||}_{\infty }=0. \end{array}$$

By Remark 8, (51) and (52) together give the desired result.

## 3. Rate of Weighted *A*-Statistical Convergence

First we define the rate of weighted *A*-statistically convergent sequence as follows.

**Definition 10 .**

Let *A* = (*a*_*n*,*k*_) be a nonnegative regular matrix and let (*a*_*k*_) be a positive nonincreasing sequence. Then, a sequence *x* = (*x*_*k*_) is weighted *A*-statistically convergent to *L* with the rate of *o*(*a*_*k*_) if for each *ϵ* > 0

(53) $$\begin{array}{c} \underset{n}{\lim}\frac{1}{a_{n}}\underset{k\in E(p,\epsilon )}{\sum }a_{n,k}=0, \end{array}$$

where

(54) $$\begin{array}{c} E\left(p,\epsilon \right)=\left\{k\in N:p_{k}\left|x_{k}-L\right|\geq \epsilon \right\}. \end{array}$$

In symbol, we will write

(55) $$\begin{array}{c} x_{k}-L=S_{A}^{\bar{N}}\text{-}o\left(a_{k}\right)\;\text{as}\;k⟶\infty . \end{array}$$

We will prove the following auxiliary result by using the above definition.

**Lemma 11 .**

Let *A* = (*a*_*n*,*k*_) be a nonnegative regular matrix. Suppose that (*a*_*k*_) and (*b*_*k*_) are two positive nonincreasing sequences. Let *x* = (*x*_*k*_) and *y* = (*y*_*k*_) be two sequences such that $x_{k}-L_{1}=S_{A}^{\bar{N}}\text{-}o(a_{k})$ and $y_{k}-L_{2}=S_{A}^{\bar{N}}\text{-}o(b_{k})$. Then,

- $\left(x_{k}-L_{1})\pm (y_{k}-L_{2})=S_{A}^{\bar{N}}\text{-}o(c_{k}\right)$,
- $\left(x_{k}-L_{1})(y_{k}-L_{2})=S_{A}^{\bar{N}}\text{-}o(c_{k}\right)$,
- $\alpha (x_{k}-L_{1})=S_{A}^{\bar{N}}\text{-}o(a_{k})$, for any scalar *α*,

where *c*_*k*_ = max⁡{*a*_*k*_, *b*_*k*_}.

**Proof**

(i) Suppose that

(56) $$\begin{array}{c} x_{k}-L_{1}=S_{A}^{\bar{N}}\text{-}o\left(a_{k}\right),\;\;y_{k}-L_{2}=S_{A}^{\bar{N}}\text{-}o\left(b_{k}\right). \end{array}$$

Given *ϵ* > 0, define

(57) E′={k∈N:pk|(xk−L1)±(yk−L2)|≥ϵ},E′′={k∈N:pk|xk−L1|≥ϵ2},E′′′={k∈N:pk|yk−L2|≥ϵ2}.

It is easy to see that

(58) $$\begin{array}{c} E^{\prime }\subset E^{\prime \prime }\cup E^{\prime \prime \prime }. \end{array}$$

This yields that

(59) $$\begin{array}{c} \frac{1}{c_{n}}\underset{k\in E^{\prime }}{\sum }a_{n,k}\leq \frac{1}{c_{n}}\underset{k\in E^{\prime \prime }}{\sum }a_{n,k}+\frac{1}{c_{n}}\underset{k\in E^{\prime \prime \prime }}{\sum }a_{n,k} \end{array}$$

holds for all *n* ∈ *N*. Since *c*_*k*_ = max⁡{*a*_*k*_, *b*_*k*_}, (59) gives that

(60) $$\begin{array}{c} \frac{1}{c_{n}}\underset{k\in E^{\prime }}{\sum }a_{n,k}\leq \frac{1}{a_{n}}\underset{k\in E^{\prime \prime }}{\sum }a_{n,k}+\frac{1}{b_{n}}\underset{k\in E^{\prime \prime \prime }}{\sum }a_{n,k}. \end{array}$$

Taking limit *n* → *∞* in (60) together with (56), we obtain

(61) $$\begin{array}{c} \underset{n}{\lim}\frac{1}{c_{n}}\underset{k\in E^{\prime }}{\sum }a_{n,k}=0. \end{array}$$

Thus,

(62) $$\begin{array}{c} \left(x_{k}-L_{1}\right)\pm \left(y_{k}-L_{2}\right)=S_{A}^{\bar{N}}\text{-}o\left(c_{k}\right). \end{array}$$

Similarly, we can prove (ii) and (iii).

Recall that the modulus of continuity of *f* in *C*[*a*, *b*] is defined by

(63) $$\begin{array}{c} \omega \left(f,\delta \right)=\sup\left\{\left|f\left(x\right)-f\left(y\right)\right|:x,y\in \left[a,b\right],\left|x-y\right|<\delta \right\}. \end{array}$$

It is well known that

(64) $$\begin{array}{c} |f\left(x\right)-f\left(y\right)|\leq \omega \left(f,\delta \right)\left(\frac{\left|x-y\right|}{\delta }+1\right). \end{array}$$

**Theorem 12 .**

Let *A* = (*a*_*n*,*k*_) be a nonnegative regular matrix. If the sequence of positive linear operators *M*_*k*_ : *C*[*a*, *b*] → *C*[*a*, *b*] satisfies the conditions

- ${||M_{k}(1;x)-1||}_{\infty }=S_{A}^{\bar{N}}\text{-}o(a_{k})$,
- $\omega (f,\lambda_{k})=S_{A}^{\bar{N}}\text{-}o(b_{k})$ with $\lambda_{k}=\sqrt{M_{k}(\varphi_{x};x)}$ and *φ*_*x*_(*y*) = (*y* − *x*)^2^,

where (*a*_*k*_) and (*b*_*k*_) are two positive nonincreasing sequences, then

(65) $$\begin{array}{c} {||M_{k}(f;x)-f(x)||}_{\infty }=S_{A}^{\bar{N}}\text{-}o\left(c_{k}\right), \end{array}$$

for all *f* ∈ *C*[*a*, *b*], where *c*_*k*_ = max⁡{*a*_*k*_, *b*_*k*_}.

**Proof**

Equation (27) can be reformed into the following form:

(66) |Mk(f;x)−f(x)|≤Mk(|f(y)−f(x)|;x)+|f(x)||Mk(1;x)−1|≤Mk(1+|y−x|δ;x)ω(f,δ)+|f(x)||Mk(1;x)−1|≤Mk(1+(y−x)2δ2;x)ω(f,δ)+|f(x)||Mk(1;x)−1|≤(Mk(1;x)+1δ2Mk(φx;x))ω(f,δ)+|f(x)||Mk(1;x)−1|≤|Mk(1;x)−1|ω(f,δ)+|f(x)||Mk(1;x)−1|+ω(f,δ)+1δ2Mk(φx;x)ω(f,δ).

Choosing $\delta =\lambda_{k}=\sqrt{M_{k}(\varphi_{x};x)}$, one obtains

(67) ||Mk(f;x)−f(x)||∞≤T||Mk(1;x)−1||∞+2ω(f,λk)+||Mk(1;x)−1||∞ω(f,λk),

where *T* = ||*f*||_*∞*_. For a given *ϵ* > 0, we will define the following sets:

(68) E1′={k∈N:pk||Mk(f,x)−f(x)||∞≥ϵ},E2′={k∈N:pk||Mk(1,x)−1||∞≥ϵ3T},E3′={k∈N:pkω(f,λk)≥ϵ6},E4′={k∈N:pkω(f,λk)||Mk(1,x)−1||∞≥ϵ3}.

It follows from (67) that

(69) $$\begin{array}{c} \frac{1}{c_{n}}\underset{k\in E_{1}^{\prime }}{\sum }a_{n,k}\leq \frac{1}{c_{n}}\underset{k\in E_{2}^{\prime }}{\sum }a_{n,k}+\frac{1}{c_{n}}\underset{k\in E_{3}^{\prime }}{\sum }a_{n,k}+\frac{1}{c_{n}}\underset{k\in E_{4}^{\prime }}{\sum }a_{n,k} \end{array}$$

holds for *n* ∈ *N*. Since *c*_*k*_ = max⁡{*a*_*k*_, *b*_*k*_}, we obtain from (69) that

(70) $$\begin{array}{c} \frac{1}{c_{n}}\underset{k\in E_{1}^{\prime }}{\sum }a_{n,k}\leq \frac{1}{a_{n}}\underset{k\in E_{2}^{\prime }}{\sum }a_{n,k}+\frac{1}{b_{n}}\underset{k\in E_{3}^{\prime }}{\sum }a_{n,k}+\frac{1}{c_{n}}\underset{k\in E_{4}^{\prime }}{\sum }a_{n,k}. \end{array}$$

Taking limit *n* → *∞* in (70) together with Lemma 11 and our hypotheses (i) and (ii), one obtains

(71) $$\begin{array}{c} \underset{n}{\lim}\frac{1}{c_{n}}\underset{k\in E_{1}^{\prime }}{\sum }a_{n,k}=0. \end{array}$$

This yields

(72) $$\begin{array}{c} {||M_{k}(f;x)-f(x)||}_{\infty }=S_{A}^{\bar{N}}\text{-}o\left(c_{k}\right). \end{array}$$

## 4. Example and the Concluding Remark

The operators *B*_*n*_ : *C*[0; 1] → *C*[0; 1] given by

(73) $$\begin{array}{c} B_{n}\left(f,x\right):=\overset{n}{\underset{k=0}{\sum }}p_{n,k}\left(x\right)f\left(\frac{k}{n}\right), \end{array}$$

where *p*_*n*,*k*_(*x*) are the fundamental Bernstein polynomials defined by

(74) $$\begin{array}{c} p_{n,k}\left(x\right)=\left(\begin{array}{c} n\\ k \end{array}\right)x^{k}{\left(1-x\right)}^{n-k} \end{array}$$

for any *x* ∈ [0,1], any *k* ∈ {0,1,…, *n*}, and any *n* ∈ *N*, are called Bernstein operators and were first introduced in [28]. Let the sequence (*A*_*n*_) be defined by *A*_*n*_ : *C*[0,1] → *C*[0,1] with *A*_*n*_(*f*, *x*) = (1 + *x*_*n*_)*B*_*n*_(*f*, *x*), where *x* = (*x*_*k*_) is a sequence defined by

(75) $$\begin{array}{c} x=\left(x_{k}\right)=\{\begin{array}{cc} \sqrt{k}, & \text{if}\;k=n^{2},\;n\in N,\\ 0, & \text{otherwise}. \end{array} \end{array}$$

That is, (*x*_*k*_) = (1,0, 0,2, 0,0, 0,0, 3,0,…, 0,4, 0,0,…). Let *p*_*k*_ = *k*  (*k* = 1,2,…) and consider a nonnegative regular matrix *A* = (*C*, 1). Then,

(76) $$\begin{array}{c} p_{k}x_{k}=\left(1,0,0,8,0,0,0,0,27,0,\ldots ,0,64,0,0,\ldots \right),\\ P_{n}=\overset{n}{\underset{k=1}{\sum }}p_{k}=\frac{n\left(n+1\right)}{2}. \end{array}$$

Since

(77) lim⁡n1Pn|{k≤Pn:pk|xk−0|≥ϵ}| ≤lim⁡n1PnPn=1n(n+1)/2=0,

(*x*_*k*_) is weighted statistically convergent to 0 but not convergent. It is not difficult to see that

(78) $$\begin{array}{c} B_{n}\left(1,x\right)=1,\;\;B_{n}\left(t,x\right)=x,\\ B_{n}\left(t^{2},x\right)=x^{2}+\frac{x-x^{2}}{n} \end{array}$$

and the sequence (*A*_*n*_) satisfies conditions (20). This yields that

(79) $$\begin{array}{c} S_{\left(C,1\right)}^{\bar{N}}\text{-lim}{||A_{n}(f,x)-f(x)||}_{\infty }=0. \end{array}$$

On the other hand, one obtains *A*_*n*_(*f*, 0) = (1 + *x*_*n*_)*f*(0), since *B*_*n*_(*f*, 0) = *f*(0), and hence

(80) $$\begin{array}{c} {||A_{n}(f,x)-f(x)||}_{\infty }\geq \left|A_{n}\left(f,0\right)-f\left(0\right)\right|=x_{n}\left|f\left(0\right)\right|. \end{array}$$

It follows that (*A*_*n*_) does not satisfy the Korovkin theorem, since (*x*_*n*_) and hence (*A*_*n*_) is not convergent. Finally, we conclude that Theorem 5 is stronger than Theorem 2.
